# A multidirectional beam steering reflector actuated by hydraulic control

**DOI:** 10.1038/s41598-019-41647-7

**Published:** 2019-03-25

**Authors:** Chao Liu, Di Wang, Qiong-Hua Wang

**Affiliations:** 1 School of Instrumentation and Optoelectronic Engineering, Beihang University, Beijing, 100191 China; 2 Beijing Advanced Innovation Center for Big Data-based Precision Medicine, Beihang University, Beijing, 100191 China

## Abstract

This paper presents a multidirectional beam steering reflector (MBSR) actuated by hydraulic control. It consists of three substrates, an elastic membrane, a magnetic base and a mirror reflector (MR). The MR is fixed on the magnetic base and covered upon the top substrate. The bottom substrate is designed with three channels for pulling in/out the liquid. When liquid volume changes, the shape of the elastic membrane changes to form a liquid piston, accordingly. The liquid piston can make the MR rotate to different directions. When a light beam irradiates the MR, it can achieve the function of beam steering in latitude and longitude, simultaneously. Our experiments show that the proposed MBSR can deflect the light beam through a maximum angle of 0~12.7° in latitude and six-directions in longitude. The MBSR has potential applications in the fields of free-space optical communications, laser detections and solar cells.

## Introduction

Precise adjustment of the light beam is critically important in an optical system. The most common way to control the beam is based on the prisms and mirror reflectors (MRs). The conventional solid prisms have a fixed geometrical shape, so it cannot show the continuous changes of the light path with the changing of refraction angle^1,2^. As for the MRs, researchers have developed several types of micro electro mechanical system (MEMS) mirrors^3–5^. The MEMS method has the advantages of fast response time, precise digital control and ease of integration. While the MEMS-based devices usually have to make channels, holes, cantilevers, membranes, cavities and other structures on micron scale, and special electronic circuit IC is also needed for sampling or driving. The common designs based on MEMS method can only achieve the functions of 1D or 2D beam steering. Besides, their fabrication relies on micro-nano processing technology. Hence, mass production is difficult due to the high cost and complex processing when they are designed in the sizes within dozens of microns. However, the beauty of MEMS is their small size, and MEMS could be easily fabricated if their size is enlarged to several millimeters.

In recent years, microfluidic technologies enable the active control of the shape and position of the liquids without bulky and complex mechanical moving parts. Due to these features, the microfluidic devices can be made more adaptable and reconfigurable to improve the optical performances. Numerous applications for beam steering have been demonstrated by the actuation mechanisms of electrowetting effect^6–14^, dielectrophoresis^15,16^, hydraulic control^17–22^, pneumatic control^23,24^ and electrically controlled elastic film^25^. The electrowetting-actuated liquid prisms have been studied intensively for the merit of easy fabrication, low power consumption and precise control^6–9^. The mechanisms of these types of liquid prisms are usually based on continually changing the tilt angle of the liquid-liquid (L-L) interface. Unfortunately, the surface type of the L-L interface is largely affected by gravity effect, leading to a divergence of the incident light. As we know, the quality of a light spot at the focal plane is directly dependent on the spatial drift of the light beam. Dielectrophoresis-actuated devices have the advantages of broadband and polarization -independent for beam steering without moving parts. But the edge interaction between the polymer dams can affect the range of the steering angle and beam quality of the device^15^. Liquid prism-like devices controlled by hydraulic and pneumatic control have formed one of the significant branches in the area of optofluidics. These liquid prisms are usually fabricated by polydimethylsiloxane (PDMS) with or without liquid filling in the hollow prism^21,22^. These prisms can be highly integrated in the microchips, but the rigid geometry provides a limitation in developing the optofluidic systems. Furthermore, these kinds of designs are often affected by mechanical vibrations picked up from the external environment.

Several types of liquid crystal (LC) beam-steering devices^26–30^ and polarization gratings devices^31–33^ have been also developed. The most important feature of LC-based designs is the fast response time which is usually within tens of milliseconds. However, the power loss and wavefront deformation which is like diffraction grating cannot be ignored.

In this paper, we design a multidirectional beam steering reflector (MBSR) actuated by hydraulic control. The motivation of the proposed approach is to realize the function of multidirectional beam steering with a simple fabrication, low cost and having a relative wide tracking angle. In this paper we use the hydraulic control method which employs the syringe pump to form the liquid elastic film pistons. Hence, it has the advantage of easy fabrication and low cost. Although the response time is slow, it has a reasonable mechanical stability. Compared with our previous works^34–37^, this work focuses on solving two main issues: one is to keep a relative high-quality beam shape steered by the reflectors and the other is to make an extended application in multidirectional beam steering. Compared with the other beam steering devices, the proposed MBSR can achieve the function of beam steering in latitude and longitude, simultaneously. Therefore, the real applications in laser scanning and ranging lidar systems can be greatly expanded.

## Mechanism and Fabrication

**Figure 1:**
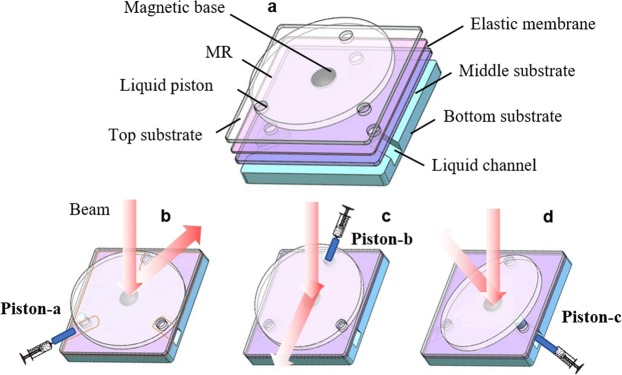
Schematic structure of the proposed MBSR and the operation mechanism. (**a**) The structure of the MBSR. (**b**) Actuating piston-a. (**c**) Actuating piston-b. (**d**) Actuating piston-c.

Figure 1 shows the schematic structure and the operation mechanism of the proposed MBSR. The bottom substrate is designed with three channels for changing the liquid volume. Two substrates with three holes and one elastic film are packaged like a sandwich. The three holes can be functioned as liquid pistons when the liquid is pulled in/out from the channels. A MR is fixed on a magnetic base and covered upon the top substrate, as shown in Fig. 1(a). When the liquid pistons are actuated, the shape of the elastic film changes accordingly. We can control the injected volume of the three liquid pistons in order to make the MR tilt to different orientations. When a light beam is incident on the MR, it can reach the function of multidirectional beam steering in longitude, as shown in Fig. 1(b–d).

**Figure 2:**
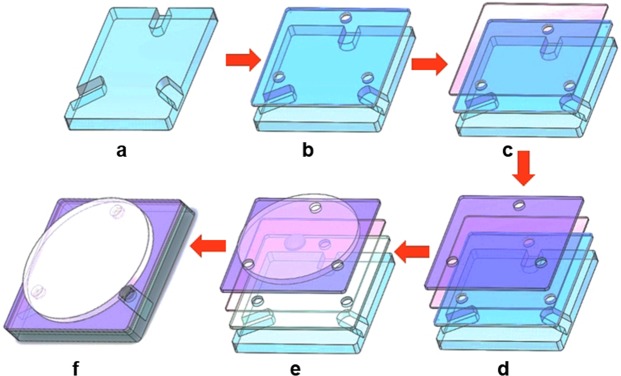
Fabrication process of the proposed MBSR. (**a**) Etching channels on the bottom substrate. (**b**) Bonding middle substrate. (**c**) Bonding elastic membrane. (**d**) Bonding top substrate. (**e**) Fixing the magnetic base and MR. (**f**) Finishing the MBSR.

Figure 2 illustrates the manufacturing flow of the proposed MBSR. Three channels are etched onto the polymethyl methacrylate (PMMA) substrate by using the soft lithography technique. The size of the bottom PMMA substrate is 19.0 mm × 19.0 mm × 4.0 mm. The width and depth of the channels are both 1.0 mm and the distance between two pairs is 120°, as shown in Fig. 2(a). Then we prepare two PMMA sheets with three holes as the middle and top substrate. The size of the two PMMA sheets are both 19.0 mm × 19.0 mm × 0.3 mm. The diameter of the hole is 2.5 mm. A polydimethylsiloxane (PDMS) elastic membrane is also prepared for forming the liquid pistons. The thickness of the elastic membrane is 200 μm (the tensile strength is 5.0 Mpa; tearing strength is 10.0 KN/m; elasticity modulus is 2.3). After that, the top substrate, the elastic membrane and the middle substrate are assembled together in a sandwich-like structure, as depicted in Fig. 2(b–d). In the end, a magnetic base is fabricated on the top substrate. The height and diameter of the magnetic base are 1.0 mm and 3.0 mm, respectively. As depicted in Fig. 2(e), an iron foil coated with silver film is functioned as the MR whose height and diameter are 150 μm and 16 mm, respectively. The weight of the iron foil is ~2.5 mg.

## Calculation, Experiment, and Discussion

### Calculations

**Figure 3:**
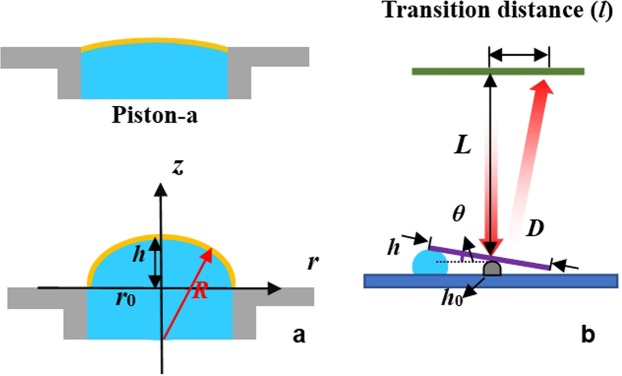
(**a**) Calculation of the curvature and liquid volume. (**b**) Calculation of the steering angle of the MR.

According to Fig. 1, when the liquid is injected into the channels, the shape of the elastic membrane changes to a convex profile. Usually, the surface configuration of the profile is like a paraboloid. Since the diameter of hole is just 2.5 mm, we can take it as a spherical surface in calculation approximately. When the fluid pump works, the volume change (Δ*V*) in the channels will force the elastic membrane to bulge outward, as depicted in Fig. 3(a). We take piston-a as an example. The radius of the piston curvature (*R*) and Δ*V* have the following relationship:

1 $${\rm{\Delta }}V=\frac{1}{3}\pi (2{R}^{2}-{r}_{0}^{2}-2R\sqrt{{R}^{2}-{r}_{0}^{2}})(2R+\sqrt{{R}^{2}-{r}_{0}^{2}}),$$

where *r*_0_ is the radius of the liquid piston. The relationship of *R* and *h* is shown in the following:

2 $$R=\frac{{h}^{2}+{r}_{0}^{2}}{2h}.$$

Hence, we can substitute Eq. (2) into Eq. (1) and calculate the relationship between the displacement (*h*) and Δ*V*.

As we know, when a mirror rotates an angle of *θ*, the reflected angle is 2*θ*. The steering angle can be calculated by the following equations:

3 $$\sin \,\theta =\frac{2(h-{h}_{0})}{D},$$

4 $$\tan \,2\theta =\frac{l}{L},$$

where *h*_0_ is the height of magnetic base, *D* is the diameter of the MR, *L* is the distance between the MR and screen, and *l* is the transition distance of the beam steering, as depicted in Fig. 3(b).

### Experiments

**Figure 4:**
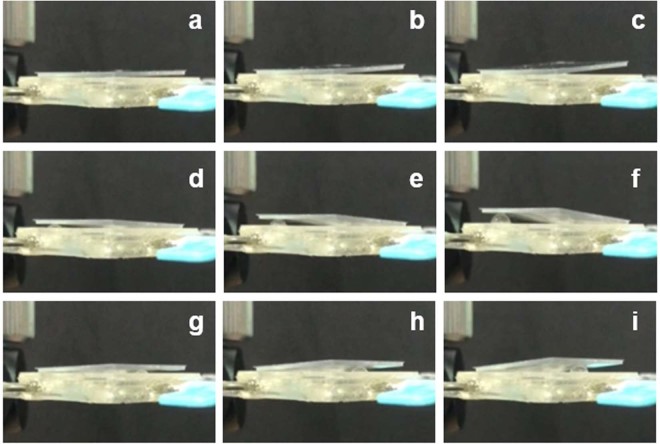
Actuating piston-a: (**a**) ΔV = 5 μl, (**b**) ΔV = 10 μl, (**c**) ΔV = 15 μl. Actuating piston-b: (**d**) ΔV = 5 μl, (**e**) ΔV = 10 μl, (**f**) ΔV = 15 μl. Actuating piston-c: (**g**) ΔV = 5 μl, (**h**) ΔV = 10 μl, (**i**) ΔV = 15 μl.

In the first principle experiment, we use a cube PMMA sheet (the height and weight are 500 μm and ~5 mg) to replace the circular MR and directly cover on the top substrate without the magnetic base. The purpose is to indicate that the liquid pistons can function well with a heavier sheet in a relative high resistance environment. In this experiment, we inject liquid (water with a density of 1.0 g/cm^3^) into the channels using a fluid pump (Longer Pump TS-1B, China). The speed of the pump is 5 μl/s. When piston-a is actuated under the liquid volume of 5 μl, 10 μl and 15 μl, respectively, the height of the piston-a changes accordingly, as shown in Fig. 4(a–c). The changes of piston-b and piston-c are the same, as depicted in Fig. 4(d–i). When the light beam irradiates the MR, it can achieve the function of beam steering. The dynamic response video of the actuated pistons is included in Media 1.

**Figure 5:**
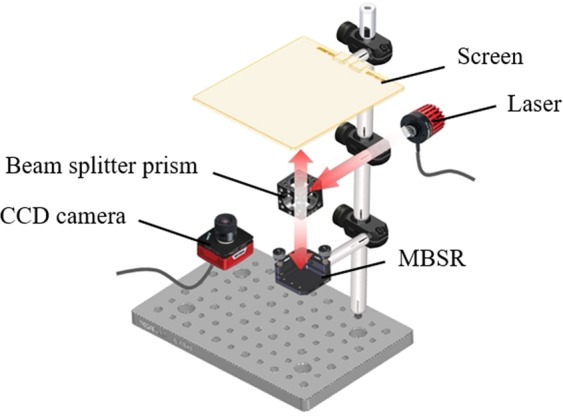
Experimental setup the MBSR.

The experimental setup consists of a beam splitter, a He-Ne laser (λ = 632.8 nm) and a CCD camera, as shown in Fig. 5. We adjust the distance (*L*) between the MR and screen to 100 mm.

**Figure 6:**
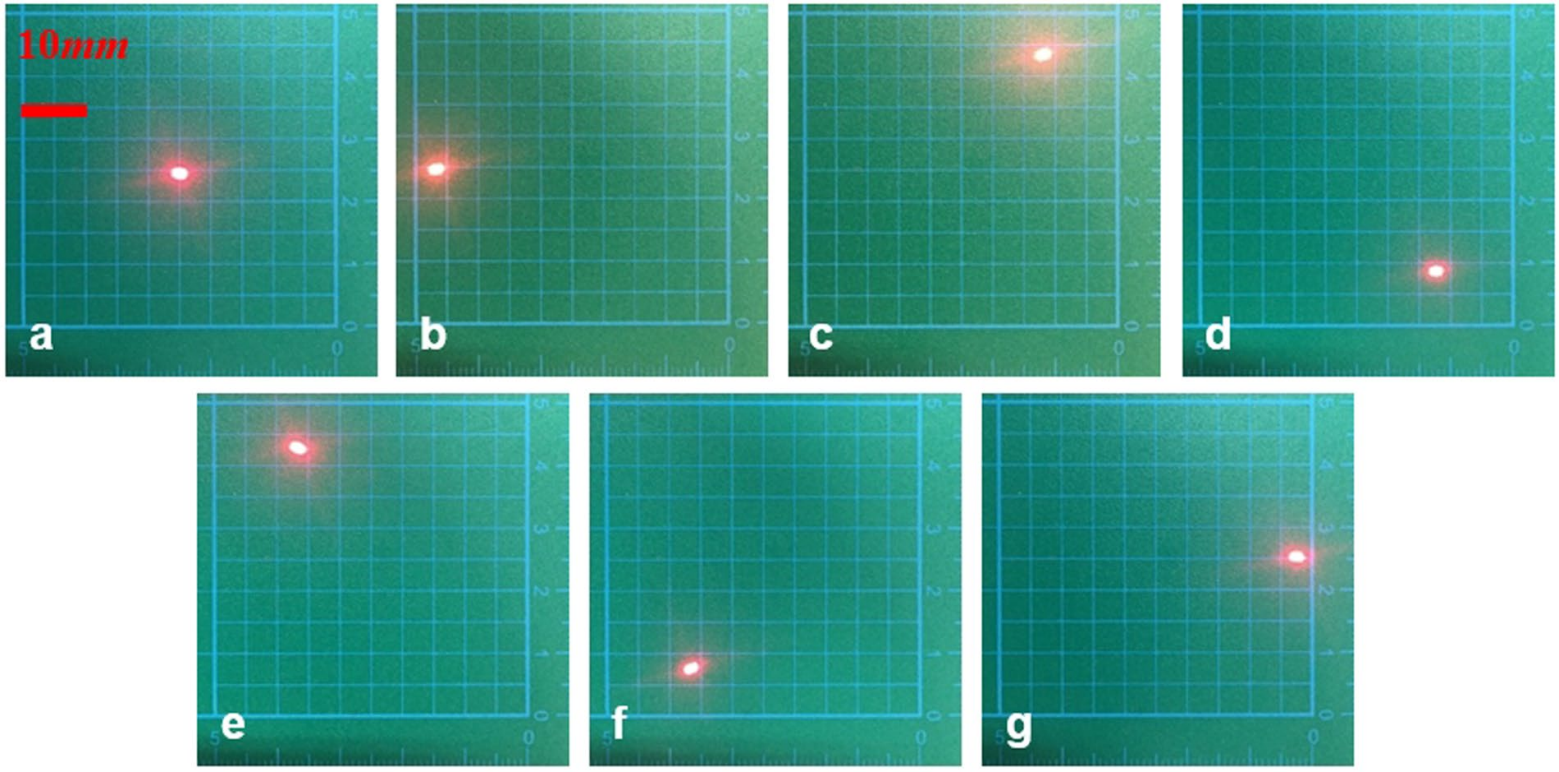
The locations of the beam spot on the screen when different liquid pistons are actuated. (**a**) Initial state. (**b**) Actuating piston-a. (**c**) Actuating piston-b. (**d**) Actuating piston-c. (**e**) Actuating piston-a + piston-b. (**f**) Actuating piston-a + piston-c. (**g**) Actuating piston-b + piston-c.

In the second experiment, we use the laser to irradiate the MR to check the laser beam steering function. In initial state, we adjust the light beam to be reflected in the center of the screen, as shown in Fig. 6(a). Then we actuate piston-a, piston-b and piston-c, successively. The maximum liquid volume change is 15 μl. The results are shown in Fig. 6(d–d). We also actuate piston-a + piston-b at the same time with the liquid volume change of 15 μl, as shown in Fig. 6(e). In this state, the laser beam can be steered another direction. The same light beam steering function towards to the other directions can be acquired when piston-a + piston-c and piston-b + piston-c are actuated, as shown in Fig. 6(f,g). From the experiments, we can draw a conclusion that the proposed MBSR can reach the function of six-directions beam steering in longitude. The dynamic response video of the beam steering is included in Media 2 by simply actuating two liquid pistons. This video just shows that the proposed device can realize the beam steering function schematically and it has a reasonable recoverability.

We have measured the transition distance actuated by liquid pistons when the MBSR is injected different volume of the liquid. The data is recorded in Table 1. The maximum transition distance under six types of actuated pistons are 22.5 mm, 20.8 mm, 21.6 mm, 20.7 mm, 22.3 mm and 22.5 mm. We put the data into Eqs 3 and 4, the beam steering angle in latitude are calculated to be 12.7°, 11.7°, 12.2°, 11.7°, 12.6° and 12.7°, respectively.

**Table 1:** Transition distance when different liquid pistons are actuated (mm).

Actuated piston	Piston-a	Piston-b	Piston-c	Piston-a Piston-b	Piston-a Piston-c	Piston-b Piston-c
Liquid volume (μl)						
5	7.1	6.6	6.5	6.6	7.2	7.0
10	16.3	15.1	15.4	15.5	16.0	16.2
15	22.5	20.8	21.6	20.7	22.3	22.5

**Figure 7:**
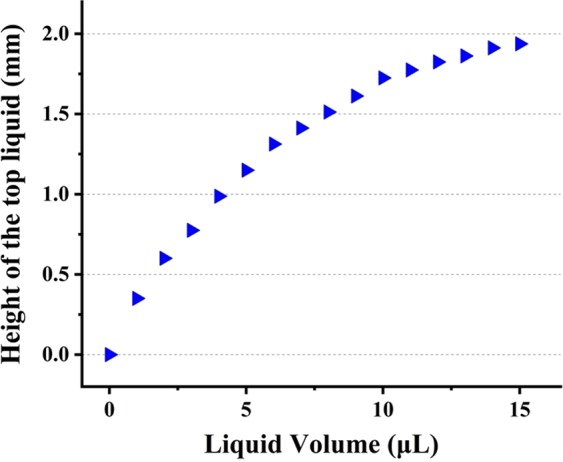
The measurement of the displacement (*h*) vs. Δ*V*.

The height of the top liquid has an influence on the steering angle of the MBSR. We take piston-a as an example and measure the displacement (*h*) and Δ*V*, as shown in Fig. 7. As we can see from Fig. 7, the maximal displacement (*h*) is ~1.9 mm.

**Figure 8:**
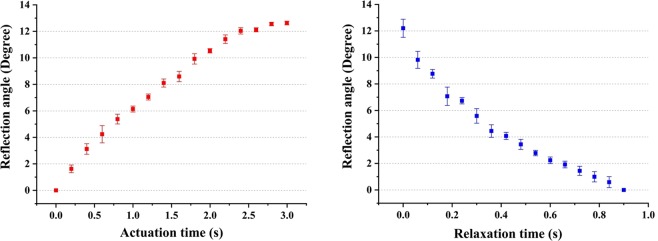
Response time of the tracking angles in the state of actuation and relaxation. (**a**) Actuation time vs. Reflection angle. (**b**) Relaxation time vs. Reflection angle.

Response time is another key parameter to measure the performance of the MBSR. We define the actuation time as the liquid volume changes from 0 ul to 5 μl when the liquid pistons are actuated. And the relaxation time is the time of 15 μl liquid volume changing from removing the fluid pump to the liquid piston recovering to its original shape with the volume changes of 15 μl. In order to prove the repeatability of the MBSR, we measured the data once a day for the duration of five days. The actuation time and relaxation time for different reflection angles are shown in Fig. 8(a,b). The measured maximal actuation time and relaxation time are 2950 ms and 900 ms, respectively.

### Discussions

Compared with the reported liquid beam steering devices, the mean advantages of this work are listed in Table 2. In addition, the MBSR can also be expanded to 360° rotatable beam steering by two means: one is to increase the number of the liquid pistons which are distributed on the substrate, uniformly; the other is to control three liquid pistons in a chronological order. For detail, the three pistons are injected with liquid in the same speed at regular intervals. When one piston reaches to its volume limitation, we pull out the liquid from this piston. Meanwhile, the other two pistons are injected with liquid continually until to the volume limitation. Using this method move in circles, the device can achieve 360° beam steering. From Fig. 8 we can see that the actuation time is relatively slow (2950 ms). That all depends on the injection speed of pump. The highest injection speed of our pump can reach 882.5 μl/ms, which means that the actuation time can achieve 17 ms. However, the fast injection speed can cause a vibration in the MBSR which has a negative influence on the mechanical stability. So we should take a tradeoff between the fast speed and mechanical stability.

**Table 2:** Comparison of several types of beam steering devices.

	Actuation	Tilt angle in longitude	Tilt angle in latitude	Shape of the steered beam	Speed time
Terrab^12^	Electrowetting	One-direction	0–4.3°	Dispersion	—
Lin^15^	Dielectrophoresis	Two-directions	0–4.0°	Dispersion	100 ms
Gou^29^	Field stress	One-direction	0–7.6°	Dispersion	10 ms
Wang^22^	Hydraulic control	One-direction	0–30°	Dispersion	50 ms
Werber^23^	Pneumatic control	One-direction	0–75°	No dispersion	—
This work	Hydraulic control	Six-directions	0°–12.7°	No dispersion	2950 ms

**Figure 9:**
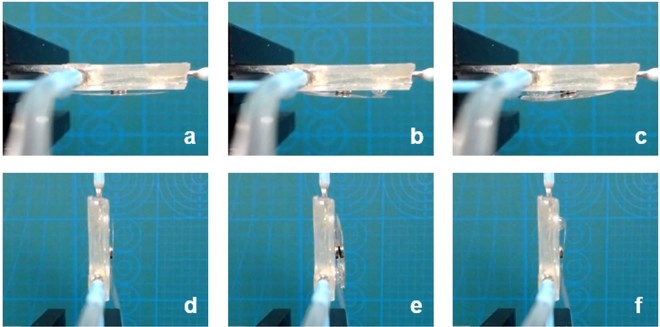
States when the MBSR is placed in opposite and vertical directions. (**a**) Initial state in opposite direction. (**b**) Actuating piston-a. (**c**) Actuating piston-b. (**d**) Initial state in vertical direction. (**e**) Actuating piston-a. (**f**) Actuating piston-b.

In order to indicate that the proposed MBSR can work in opposite and vertical directions, we do another experiment. We reinstall the MBSR and make it in opposite and vertical positions. The initial state and piston-actuated states in opposite direction are shown in Fig. 9(a–c), respectively. The initial state and piston-actuated states in vertical direction are shown in Fig. 9(d,e), respectively. The dynamic video is also included in Media 3.

## Conclusions

In this paper, we report an MBSR actuated by hydraulic control. By fabricating three liquid pistons, the MBSR can have a six-directions beam steering function in longitude. Our experiments show that the MBSR can also achieve beam steering within 12.7° in latitude. The measured actuation time and relaxation time are 2950 ms and 900 ms, respectively. The proposed MBSR can be expanded to 360° rotatable beam steering and work in opposite and vertical directions. The MBSR has potential applications in the fields of free-space optical communications, laser detections, and solar cells.

## Supplementary information

Media-1

Media-2

Media-3
